# Factors associated with readmission to the hospital within 30 days in patients with inflammatory bowel disease

**DOI:** 10.1371/journal.pone.0182900

**Published:** 2017-08-24

**Authors:** Dejan Micic, John N. Gaetano, Jonah N. Rubin, Russell D. Cohen, Atsushi Sakuraba, David T. Rubin, Joel Pekow

**Affiliations:** 1 Department of Internal Medicine, Section of Gastroenterology, Hepatology and Nutrition, University of Chicago, Chicago, Illinois, United States of America; 2 Department of Internal Medicine, Section of Hospital Medicine, University of Chicago, Chicago, Illinois, United States of America; University Hospital Llandough, UNITED KINGDOM

## Abstract

**Background:**

Management of inpatients with inflammatory bowel disease (IBD) requires increasing resources. We aimed to identify factors associated with hospital readmissions among individuals with IBD.

**Materials & methods:**

We collected data from the Healthcare Cost and Utilization Project Nationwide Readmissions Database 2013. We identified individuals with index hospitalizations for IBD. Patient-specific factors, comorbidities and hospitalization characteristics were extracted for the index hospitalization. We performed logistic regression modeling to create adjusted odds ratios (ORs) for 30-day hospital readmission. Subgroup analysis was performed based on disease type and performance of surgery.

**Results:**

We analyzed a total of 55,942 index hospital discharges; 3037 patients (7.0%) were readmitted to the hospital within 30 days. Increasing patient age (> 65: OR: 0.45; 95% CI 0.39–0.53) was associated with a decreased risk of readmission, while a diagnosis of Crohn’s disease (OR: 1.09; 95% CI 1.00–1.18) and male sex (OR: 1.16; 95% CI 1.07–1.25) were associated with an increased risk of readmission. The comorbidities of smoking (OR: 1.09; 95% CI 1.00–1.19), anxiety (OR: 1.17; 95% CI 1.01–1.36) and opioid dependence (OR: 1.40; 95% CI 1.06–1.86) were associated with an increased risk of 30-day readmission. Individual hospitalization characteristics and disease complications were significantly associated with readmission. Performance of a surgery during the index admission was associated with a decreased risk of readmission (OR: 0.57; 95% CI 0.33–0.96).

**Conclusion:**

Analyzing data from a US publicly available all-payer inpatient healthcare database, we identified patient and hospitalization risk factors associated with 30-day readmission. Identifying patients at high risk for readmission may allow for interventions during or after the index hospitalization to decrease this risk.

## Introduction

Crohn’s disease (CD) and ulcerative colitis (UC) are chronic inflammatory bowel diseases (IBD) that affect approximately 1.6 million people in the United States [1]. The incidence and prevalence of inflammatory bowel disease is rising worldwide with increased trends in the rates of hospitalization and resource utilization [1]. A meta-analysis of population based studies demonstrated pooled risks of surgery at 1 year and 5 years for CD to be 14.3% and 27.7% and for UC 4.1% and 9.9%, respectively, with a decreased trend in surgical rates over the past 6 decades [2]. This decrease in surgical rates is hypothesized to be related to increased exposure to and use of biologic therapies as CD-related hospitalizations and intra-abdominal surgeries were decreased with infliximab maintenance therapy in the pivotal ACCENT I and II studies [3, 4].

Along with a rising incidence of hospitalizations in IBD, inpatient healthcare costs remain a significant burden of healthcare utilization. Hospitalizations contribute to a substantial component of care among individuals with CD and surgery accounts for the greatest proportion of inpatient costs [5]. In a previous analysis using an administrative insurance claims database, hospitalizations accounted for 31.4% and 37.6% of total costs for CD and UC, respectively. Anti-TNF therapy accounted for 29.8% of healthcare costs and surgical hospitalizations accounted for 12.4% of costs [5, 6].

Previous studies and a meta-analysis have identified 9–50% of acute care readmissions as preventable, with the wide range indicating differences in definitions of preventable readmission and quality of care [7, 8]. In an assessment of readmissions among nine Veterans Affairs Medical Centers, in which 33–34% of readmissions were judged as preventable, the most common reason for preventable readmission was a lack of physician assessment and/or change in therapy in the two weeks prior to admission [9].

The majority of studies examining readmission risk in IBD have either been single-center studies or have focused on surgical patients [10, 11]. As such, understanding risk factors such as patient specific comorbidities, socioeconomic factors and hospitalization factors leading to readmissions among a large IBD cohort could allow for guided quality improvement interventions aimed at decreasing readmission rates. Therefore, the aim of this study was to identify factors associated with readmission utilizing a novel national survey database.

## Materials & methods

### Data source

The Healthcare Cost and Utilization Project (HCUP) Nationwide Readmissions Database (NRD) 2013, sponsored by the Agency for Healthcare Research and Quality (AHRQ) is part of a family of publicly available all-payer inpatient healthcare databases in the United States. The NRD is drawn from 21 HCUP State Inpatient Databases (SID) containing patient linkage numbers that can be used to track persons across hospitals within a state. The NRD 2013 contains data from approximately 14 million discharges, accounting for 49.1% of all U.S. hospitalizations, including all non-federal short-term general and subspecialty hospitals, public hospitals and academic medical centers [12].

### Definition of variables

Demographic information was obtained on age, sex, insurance type, and median income for patient ZIP code. Each data entry includes a unique patient identifier, type of index admission (emergency, urgent or elective), hospital teaching status, hospital volume, primary ICD-9-CM diagnosis with up to 24 secondary diagnoses, ICD-9-CM procedure codes (up to 15), primary insurance payers, total hospital charges, and length of stay [13]. Information regarding hospital volume, severity of illness and comorbidity status is listed in S1 Table.

### Definition of study groups

Our primary case group included adult patients with (a) a primary discharge diagnosis of CD (ICD-9-CM 555.x) or UC (ICD-9-CM 556.x); or (b) a primary diagnosis of an IBD–related complication and a secondary diagnosis of CD or UC as defined in previous investigations [13, 14]. IBD-related complications and systemic complications of IBD that were included as primary diagnoses are listed in S1 Table [14]. Patients that died during the index admission (n = 655) were excluded from the analysis.

### Outcomes

The primary outcome was all-cause hospital readmission within 30 days of the discharge date of the index hospitalization. We excluded readmissions characterized as elective. Since the database captured admission purely on a calendar year basis (ie. January 1^st^ through December 31^st^) without a linkage to the previous or following year, index hospitalization discharges occurring in the month of December were excluded from the analysis. Among individuals with multiple readmissions, only the first index hospitalization and readmission were included in the analysis. Once it was determined which individuals had subsequent 30-day readmissions, and time to subsequent readmission was captured, all repeat readmissions were removed from the dataset, leaving a dataset of only first index hospitalizations, allowing for the assumption of independence between observations to be met for the regression model.

### Statistical analysis

Continuous variables were summarized using means and standard deviations. The use of proportions was used for categorical variables. Age was utilized as a categorical value as provided in the NRD. Comparison between groups (readmitted vs. not readmitted) was performed using the Student’s t-test for continuous variables and the Chi-square for categorical variables. Logistic regression was utilized for a multivariate analysis of risk factors for 30-day readmission. The factors significant in univariate analyses at *P* ≤ 0.05 were included in the final multivariate models to produce odds ratios and 95% confidence intervals (CI) for 30-day readmissions. The predictors were considered significant at *P* < 0.05 in the final model. Subgroup analysis was performed among individuals with CD, UC, and in patients undergoing surgery during the index hospitalization. Multiple linear regression models were utilized to develop area under the receiver operator curves (AUROC). Statistical analyses performed using SPSS Version 22 (IBM Corporation, Armonk, NY, USA). GraphPad Prism (GraphPad Software, Inc, La Jolla, CA, USA) was used to create figures.

## Results

A total of 55,942 hospital discharges with a diagnosis of IBD met our inclusion criteria: 35,788 (64.0%) with CD and 20,154 (36.0%) with UC. Of these, 43,680 (78.1%) represented index hospitalizations, while 12,262 (21.9%) were repeat readmissions from a previously captured hospitalization (Table 1).

**Table 1:** Demographic, patient and hospital characteristics.

Characteristic	Total (n = 55,942)	Crohn’s disease (n = 35,788)	Ulcerative colitis (n = 20,154)	*p*-value
Mean age (years)	47.8	45.8	51	<0.001
**Age group (years)**				
18–35	18,528 (33.1%)	12,745 (35.6%)	5783 (28.7%)	<0.001
36–50	13,626 (24.4%)	9396 (26.3%)	4230 (21.0%)	
51–65	12,359 (22.1%)	7668 (21.4%)	4691 (23.3%)	
>65	11,429 (20.4%)	5979 (16.7%)	5450 (27.0%)	
**Sex**				
Male	25,050 (44.9%)	15,711 (43.9%)	9349 (46.4%)	<0.001
Female	30,862 (55.2%)	20,077 (56.1%)	10,805 (53.6%)	
**APR-DRG Risk of Mortality**				
Minor	36,193 (64.7%)	23,552 (65.8%)	12,641 (62.7%)	<0.001
Moderate	11,800 (21.1%)	7569 (21.1%)	4231 (21.0%)	
Major	6282 (11.2%)	3729 (21.1%)	4231 (21.0%)	
Extreme	1666 (3.0%)	938 (2.6%)	728 (3.6%)	
Smoking	14,549 (26.0%)	10,412 (29.1%)	4137 (20.5%)	<0.001
Depression	9710 (17.4%)	6621 (18.5%)	3089 (15.3%)	<0.001
Anxiety	7387 (13.2%)	5063 (14.1%)	2324 (11.5%)	<0.001
Depression and anxiety	3649 (6.5%)	2558 (7.1%)	1091 (5.4%)	<0.001
Opioid dependence	880 (1.6%)	676 (1.9%)	204 (1.0%)	<0.001
Cannabis dependence	713 (1.3%)	539 (1.5%)	174 (0.9%)	<0.001
Length of stay (mean days)	5.5	5.3	5.9	<0.001
Total charges (mean USD)	43,390	41,345	47,060	<0.001
**Primary payer**				
Medicare	17,531 (31.1%)	10,703 (29.9%)	6648 (33.0%)	<0.001
Medicaid	8078 (14.5%)	5606 (15.7%)	2472 (12.3%)	
Private	23, 546 (42.1%)	14, 898 (41.7%)	8648 (43.0%)	
Self-pay	3812 (6.8%)	2598 (7.3%)	1214 (6.0%)	
No charges	611 (1.1%)	418 (1.2%)	193 (1.0%)	
Other	2478 (4.4%)	1519 (4.2%)	959 (4.8%)	
**Median income quartiles for patient’s ZIP code**				
Quartile 1 (lowest income)	12, 947 (23.5%)	8538 (24.2%)	4409 (22.2%)	<0.001
Quartile 2	14,082 (25.5%)	9225 (26.2%)	4857 (24.5%)	
Quartile 3	14,303 (25.9%)	9065 (25.7%)	1214 (6.0%)	
Quartile 4 (highest income)	13,795 (25.0%)	8447 (23.9%)	5348 (26.9%)	
**Teaching status of hospital**				
Metropolitan teaching	22, 458 (40.1%)	14,210 (39.7%)	8248 (40.9%)	<0.001
Metropolitan non-teaching	29, 429 (52.6%)	18, 848 (52.7%)	10, 581 (52.5%)	
Non-metropolitan	4055 (7.2%)	2730 (7.6%)	1325 (6.6%)	
**Hospital volume**				
Low	5757 (10.3%)	3670 (10.3%)	2087 (10.4%)	0.344
Medium	13, 378 (23.9%)	8629 (24.1%)	4749 (23.6%)	
High	36,807 (65.8%)	23,489 (65.6%)	13,318 (66.1%)	
**Disease complications**				
Intraabdominal fistula or abscess	4397 (7.9%)	4397 (12.3%)	-	
Perianal fistula or abscess	1309 (2.3%)	1309 (3.7%)	-	
Stricture	6348 (11.3%)	5565 (15.5%)	783 (3.9%)	<0.001
Bowel obstruction	3808 (6.8%)	2766 (7.7%)	1042 (5.2%)	<0.001
Gastrointestinal bleeding	3433 (6.1%)	1881 (5.3%)	1552 (7.7%)	<0.001
*Clostridium difficile* colitis	3926 (7.0%)	1679 (4.7%)	2247 (11.1%)	<0.001
Hypovolemia	9445 (16.9%)	5481 (15.3%)	3964 (19.7%)	<0.001
Electrolyte disturbance	16,274 (29.1%)	9740 (27.2%)	6534 (32.4%)	<0.001
Anemia	5733 (10.2%)	3190 (8.9%)	2543 (12.6%)	<0.001
Malnutrition	5950 (10.6%)	3677 (10.3%)	2273 (11.3%)	<0.001
**Hospitalization characteristics**				
Lower endoscopy	12,266 (21.9%)	5773 (16.1%)	6493 (32.2%)	<0.001
Abdominal CT scan	2218 (4.0%)	1419 (4.0%)	799 (4.0%)	0.997
Blood transfusion	6335 (11.3%)	3294 (9.2%)	3041 (15.1%)	<0.001
Parenteral nutrition	2541 (4.5%)	1840 (5.1%)	701 (3.5%)	<0.001
Small bowel resection	1324 (2.4%)	1159 (3.2%)	165 (0.8%)	<0.001
Colectomy (partial or total)	2828 (5.1)	2117 (5.9%)	711 (3.5%)	<0.001
Any surgery performed	3722 (6.7%)	2891 (8.1%)	831 (4.1%)	<0.001
Elective surgery	1807 (3.2%)	1358 (3.5%)	549 (2.7%)	<0.001
Urgent surgery	1495 (2.7%)	1298 (3.6%)	197 (1.0%)	<0.001
Index admission, no subsequent readmission	35,935 (64.2%)	21,387 (59.8%)	14,548 (72.2%)	<0.001
Index admission, subsequently readmitted	7745 (13.8%)	5384 (15.0%)	2361 (11.7%)	
Repeat readmission	12262 (21.9%)	9017 (25.2%)	3245 (16.1%)	

### Hospitalization and patient characteristics

Table 1 presents the demographic, patient characteristics and hospital characteristics for the included index hospital discharges, as well as the differences between individuals with UC and CD. Mean patient age was 47.8 years and patients were more commonly female (55.2% vs 44.8%; *P* <0.001). Compared to UC, patients with CD were more likely to be smokers (29.1% vs 20.5%; *P* < 0.001) and have a discharge diagnosis of bowel obstruction (7.7% vs 5.2%; *P* < 0.001). Patients with UC were more likely to have a hospitalization with *Clostridium difficile* colitis (11.1% vs 4.7%; *P* < 0.001), malnutrition (11.3% vs 10.3%; *P* < 0.001), and electrolyte disturbance (32.4% vs 27.2%; *P* < 0.001).

### Univariate and multivariate analysis of 30-day readmission

**Fig 1:**
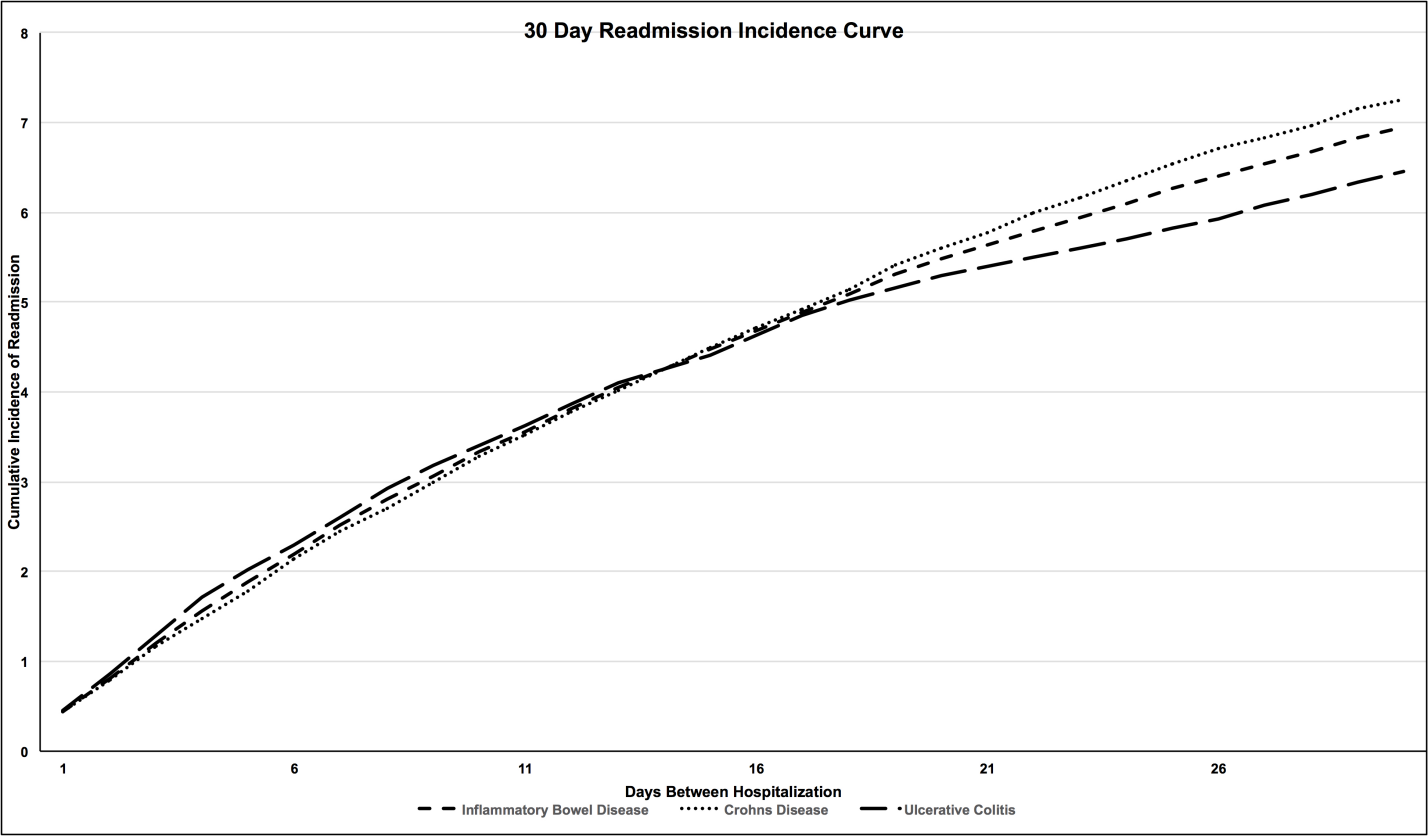
Cumulative incidence of readmission for IBD, CD and UC. (y-axis: percent of readmission when compared to the total population).

The 12,262 hospitalizations captured as repeat readmissions were excluded from this portion of the analysis, leaving 43,680 index hospitalizations for analysis of risk factors for readmission. In this cohort, 3037 (7.0%) patients had a 30-day readmission. Cumulative incidence curves for readmission are demonstrated in Fig 1. Factors associated with readmission on univariate analysis and multivariate analysis are listed in Table 2. In the multivariate model, factors associated with a decreased risk of readmission included increasing patient age (36–50: OR: 0.75; 95% CI 0.68–0.82; 51–65: OR: 0.59; 95% CI 0.53–0.66; > 65: OR: 0.45; 95% CI 0.39–0.53) when compared to the age category 18–35. A diagnosis of CD (OR: 1.09; 95% CI 1.00–1.18), and male sex (OR: 1.16; 95% CI 1.07–1.25) were associated with an increased risk of readmission. The comorbidities of smoking (OR: 1.09; 95% CI 1.00–1.19), anxiety (OR: 1.17; 95% CI 1.01–1.36) and opioid dependence (OR: 1.40; 95% CI 1.06–1.86) were associated with an increased risk of 30-day readmission. Self–paying patients (OR: 0.83; 95% CI 0.69–0.99) and those with private insurance (OR: 0.78; 95% CI 0.69–0.89) were less likely to have a readmission when compared to individuals with Medicare. Higher quartiles of household income (> $64,000; OR: 0.88; 95% CI 0.79–0.98) were also associated with a decreased rate of readmission when compared to the lowest quartile of income ($1-$37,999). Hospitalization in a metropolitan non-teaching hospital and a non-metropolitan hospital was associated with a decreased risk of readmission when compared to a metropolitan teaching hospital: non-teaching (OR: 0.92; 95% CI 0.85–1.00), non-metropolitan (OR: 0.75; 95% CI 0.64–0.89). Disease-associated complications associated with readmission risk are listed in Table 2. Performance of a surgery during the index admission was associated with a decreased risk of readmission (OR: 0.57; 95% CI 0.33–0.96).

**Table 2:** IBD risk factors for 30-day readmission.

Characteristic	Not readmitted within 30 days (n = 40,643)	Readmitted within 30 days (n = 3037)	Univariate *p*-value	Multivariate OR (95% CI)
Mean age (years)	49.3	44.8		
Crohn’s disease	24,826 (61.1%)	1,944 (64.0%)	0.001	**1.09 (1.00–1.18)**
Ulcerative colitis	15,817 (38.9%)	1,091 (36.0%)	0.001	**Ref**
**Age group (years)**				
18–35	12.053 (29.7%)	1,207 (29.8%)	<0.001	**Ref**
36–50	9.719 (23.9%)	736 (24.3%)	0.67	**0.75 (0.68–0.82)**
51–65	9,582 (23.6%)	592 (19.5%)	<0.001	**0.59 (0.53–0.66)**
>65	9,289 (22.9%)	500 (16.5%)	<0.001	**0.45 (0.39–0.53)**
**Sex**				
Male	17,932 (44.1%)	1,429 (47.1%)	0.002	**1.16 (1.07–1.25)**
Female	22,711 (55.9%)	1,606 (52.9%)	0.002	**Ref**
**APR-DRG Risk of Mortality**				
Minor	26,114 (64.3%)	2,007 (66.1%)	0.037	
Moderate	8,602 (21.2%)	638 (21.0%)	0.852	
Major	4,678 (11.5%)	331 (10.9%)	0.314	
Extreme	1,248 (3.1%)	59 (1.9%)	<0.001	
Smoking	10,300 (25.3%)	851 (28.1%)	0.001	**1.09 (1.00–1.19)**
Depression	6,498 (16.0%)	557 (18.4%)	0.001	**NS**
Anxiety	4,854 (11.9%)	437 (14.4%)	<0.001	**1.17 (1.01–1.36)**
Depression and anxiety	2,342 (5.8%)	222 (7.3%)	<0.001	**NS**
Opioid dependence	453 (1.1%)	60 (2.0%)	<0.001	**1.40 (1.06–1.86)**
Cannabis dependence	474 (1.2%)	48 (1.6%)	0.042	**NS**
Weekend admission	8,488 (20.9%)	641 (21.1%)	0.758	
Length of stay (mean days)	5.4	5.8		
Total charges (mean USD)	43,230	42,193		
**Primary payer**				
Medicare	12,912 (31.8%)	860 (28.4%)	<0.001	**Ref**
Medicaid	4,878 (12.0%)	509 (16.8%)	<0.001	**NS**
Private	17,979 (44.3%)	1,258 (41.5%)	0.003	**0.78 (0.69–0.89)**
Self-pay	2,653 (6.5%)	223 (7.4%)	0.079	**0.83 (0.69–0.99)**
No charges	404 (1.0%)	41 (1.4%)	0.059	**NS**
Other	1,769 (4.4%)	141 (4.6%)	0.446	**NS**
**Median income quartiles for patient’s ZIP code**				
Quartile 1 (lowest income)	8,914 (22.3%)	762 (25.5%)	<0.001	**Ref**
Quartile 2	10,195 (25.5%)	756 (25.3%)	0.83	**0.90 (0.81–1.0)**
Quartile 3	10,494 (26.2%)	750 (25.1%)	0.178	**0.88 (0.79–0.98)**
Quartile 4 (highest income)	10,429 (26.1%)	722 (24.1%)	0.023	**0.88 (0.79–0.98)**
**Teaching status of hospital**				
Metropolitan teaching	20,887 (51.4%)	1,654 (54.5%)	0.001	**Ref**
Metropolitan non-teaching	16,563 (40.8%)	1,189 (39.2%)	0.088	**0.92 (0.85–1.0)**
Non-metropolitan	3,193 (7.9%)	192 (6.3%)	0.002	**0.75 (0.64–0.89)**
**Hospital volume**				
Low	4,400 (10.8%)	286 (9.4%)	0.016	
Medium	9,685 (23.8%)	758 (25.0%)	0.153	
High	26,558 (65.3%)	1,991 (65.6%)	0.774	
**Disease complications**				
Intraabdominal fistula or abscess	2,830 (7.0%)	274 (9.0%)	<0.001	**1.32 (1.15–1.52)**
Perianal fistula or abscess	866 (2.1%)	75 (2.5%)	0.213	
Stricture	4,542 (11.2%)	310 (10.2%)	0.104	
Bowel obstruction	2,896 (7.1%)	173 (5.7%)	0.003	**NS**
Gastrointestinal bleeding	2,528 (6.2%)	183 (6.1%)	0.675	
*Clostridium difficile* colitis	2,562 (6.3%)	220 (7.2%)	0.04	**NS**
Hypovolemia	6,719 (16.5%)	586 (19.3%)	<0.001	**1.23 (1.11–1.35)**
Electrolyte disturbance	11,505 (28.3%)	941 (31.3%)	0.001	**1.16 (1.06–1.26)**
Anemia	4,023 (9.9%)	334 (11.0%)	0.05	**NS**
Malnutrition	3,750 (9.2%)	393 (13.0%)	<0.001	**1.37 (1.22–1.54)**
**Hospitalization characteristics**				
Lower endoscopy	9,685 (23.8%)	745 (24.6%)	0.371	
Abdominal CT scan	1,649 (4.1%)	110 (3.6%)	0.242	
Blood transfusion	4,438 (10.9%)	379 (12.5%)	0.008	**1.19 (1.06–1.34)**
Small bowel resection	1,054 (2.6%)	39 (1.3%)	<0.001	
Colectomy (partial or total)	2,278 (5.6%)	107 (3.5%)	<0.001	
Any surgery performed	3,004 (7.4%	127 (4.2%)	<0.001	**0.57 (0.33–0.96)**
Elective surgery	1,741 (4.3%)	66 (2.2%)	<0.001	**NS**

### Univariate and multivariate analysis of secondary outcomes

**Fig 2:**
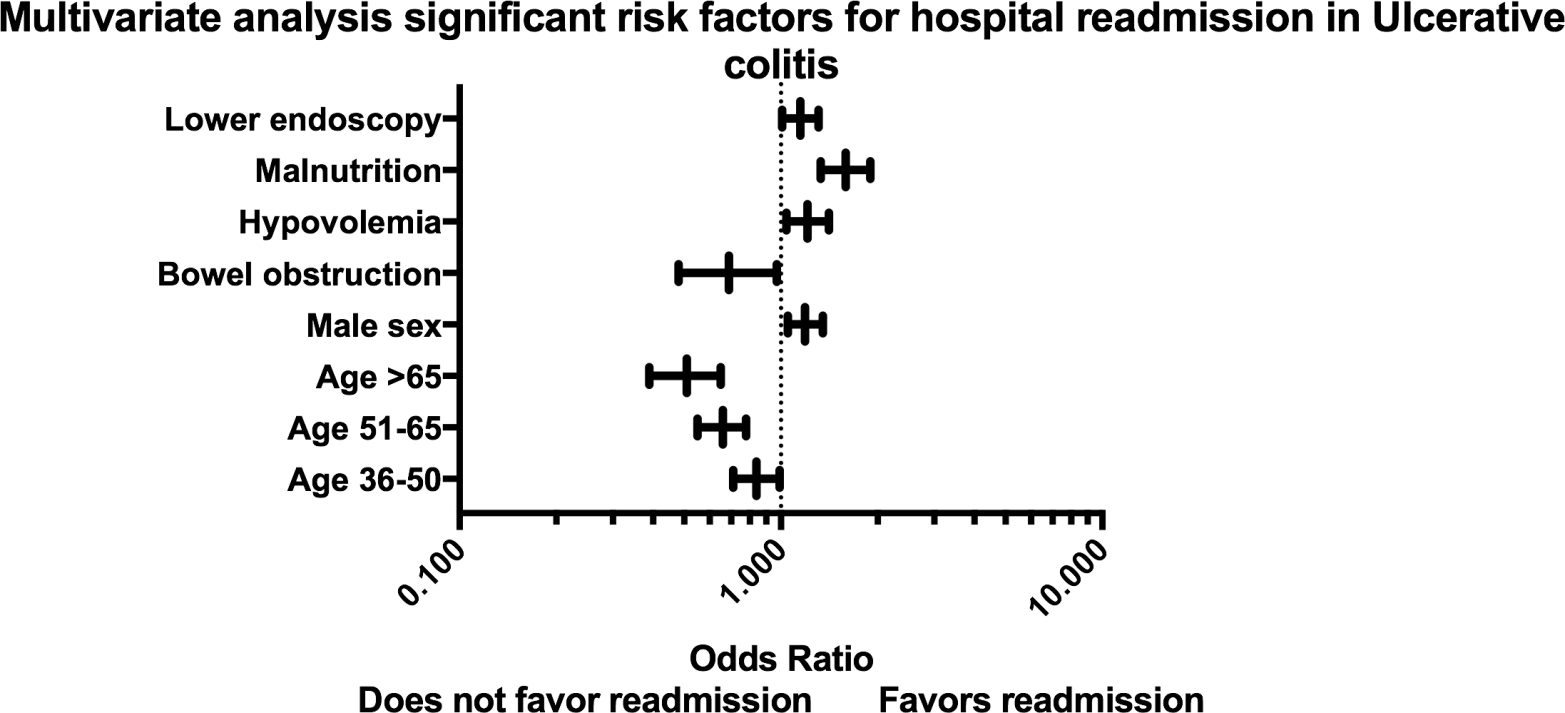
Forest plot of significant factors on multivariate analysis for 30-day readmission among individuals with ulcerative colitis.

**Fig 3:**
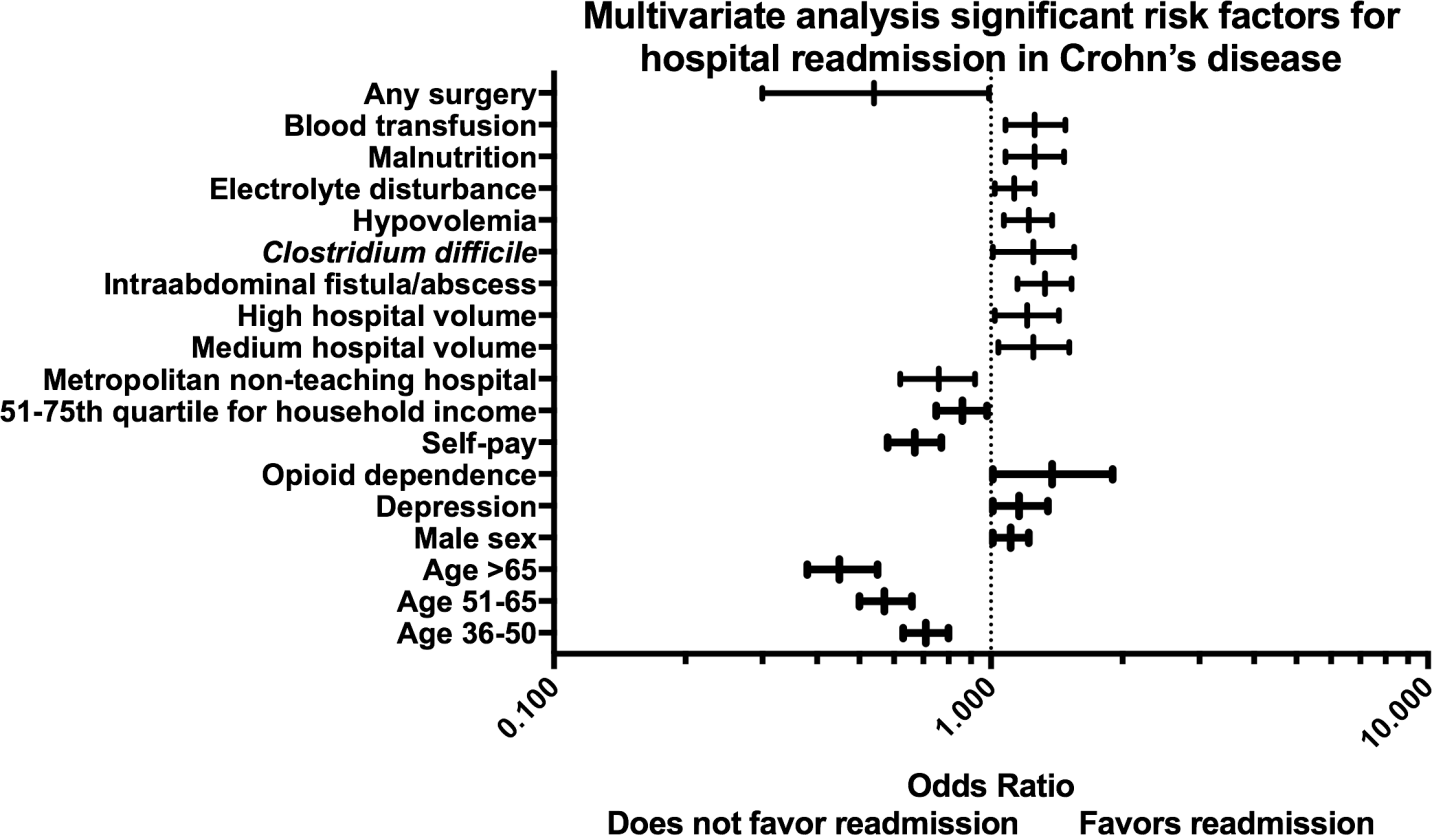
Forest plot of significant factors on multivariate analysis for 30-day readmission among individuals with Crohn’s disease.

We performed subgroup analyses to assess for risk factors associated with 30-day readmission among individuals with UC (S2 Table) and CD (S3 Table). Significant factors in the multivariate analysis associated with 30-day readmission are demonstrated for UC (Fig 2) and CD (Fig 3). Patient comorbidities or hospital characteristics were not associated with readmission among individuals with UC. Among patients with CD with private insurance (OR: 0.67; 95% CI 0.58–0.77) and those in the 51^st^-75^th^ quartile for household income (OR: 0.86; 95% CI 0.75–0.98) had a decreased risk of readmission. In UC, patients with hypovolemia (OR: 1.21; 95% CI 1.04–1.41) and malnutrition (OR: 1.59; 95% CI 1.33–1.90) during the index hospitalization had an increased risk of readmission. In subjects with CD, disease complications including: intraabdominal fistula or abscess (OR: 1.33; 95% CI 1.15–1.53), *C*. *difficile* colitis (HR: 1.25; 95% CI 1.01–1.505), hypovolemia (OR: 1.22; 95% CI 1.07–1.38), electrolyte disturbances (OR: 1.13; 95% CI 1.02–1.26), malnutrition (OR: 1.26; 95% CI 1.08–1.47) and requirement for blood transfusion (OR: 1.26; 95% CI 1.08–1.48) were all associated with an increased risk of readmission. In CD performance of surgery (OR: 0.54; 95% CI 0.3–0.99) was associated with a decreased risk of readmission.

### Univariate and multivariate analysis of patients undergoing surgical procedure

Patients undergoing surgical procedures during the index hospitalization were separately analyzed. One hundred twenty-seven (4.2%) patients were readmitted within 30 days. Univariate risk factors for readmission are listed in S4 Table. In the multivariate model, only hypovolemia (OR: 1.86; 95% CI 1.07–3.22), anemia (OR: 1.90; 95% CI 1.07–3.38), malnutrition (OR: 1.56; 95% CI 1.01–2.42), performance of a blood transfusion (OR: 1.96; 95% CI 1.27–3.02) and performance of a colectomy (OR: 1.84; 95% CI 1.30–3.0) during the index hospitalization were associated with an increased risk of readmission (S4 Table).

### Predictive modeling

A predictive model of all IBD patients including opioid dependence, malnutrition, age > 65 and undergoing surgery had an AUROC of 0.58 and an optimal cut off of 0.07 with a sensitivity of 82% and specificity of 26% for a readmission event. (S1 Fig). In CD, the combination of opioid dependence, cannabis dependence, malnutrition and undergoing surgery produced an AUROC of 0.54 with an optimal cut off of 0.09 with a sensitivity of 15% and specificity of 91% (S2 Fig). In UC, age > 65, bowel obstruction, malnutrition and undergoing surgery produced an AUROC of 0.59 with an optimal cut off of 0.06 with a sensitivity of 80% and a specificity of 31% for a readmission event (S3 Fig).

## Discussion

IBD remains a significant healthcare burden, and hospitalizations account for a substantial proportion of total healthcare costs in this population [6]. Understanding patient and hospital specific factors leading to readmission can identify potentially modifiable risk factors, allowing for guided quality improvement interventions aimed at decreasing readmission rates. Our nationwide analysis of hospital discharges provides important insights into factors related to readmissions among individuals with IBD. This study found that 7.0% of patients with IBD are readmitted during a 30-day period. In addition, we identified patient factors, hospitalization characteristics and disease complications associated with 30-day readmissions. Significant findings include decreased rates of readmissions among female and older patients. Readmission rates were also lower among individuals with private insurance, self-paying patients, as well as those included in higher estimated quartiles of household income. Readmission rates were higher among individuals with a diagnosis of anxiety and opioid dependence and the comorbidity smoking on the index hospitalization. The primary disease-associated complications associated with increased rates of readmission included hypovolemia, malnutrition and requirement for a blood transfusion during an index hospitalization.

Previous studies have attempted to identify factors related to readmission in IBD. Nguyen *et al*, analyzed the Canadian Institute for Health Information Discharge Abstract databases including 26,403 patients hospitalized for IBD [15]. Patients with a diagnosis of UC and increasing patient age were associated with a decreased risk of readmission. The performance of a bowel resection during the index hospitalization was also associated with a decreased risk of readmission, as was hospitalization in the highest volume IBD centers. A second Canadian study utilizing the Statistics Canada Person Oriented Information Database demonstrated that time to readmission varied according to province of patient residence and for IBD patients admitted for operation of incision, excision, and anastomosis of the intestine, who had a lower readmission rate than those admitted for other gastrointestinal surgeries or those admitted without surgery [16]. The performance of abdominal surgery during the index hospitalization has the potential to decrease subsequent readmissions, which is not surprising as one can achieve a surgically induced remission. This is in agreement with a Markov model highlighting the longest disease remissions induced by surgery in patients with CD [17].

Two single-center studies from the United States have also assessed for factors associated with readmission in IBD. Hazratjee *et al*. created a predictive model of 30-day readmission for individuals with IBD admitted to an inpatient gastroenterology service [18]. A predictive model for hospital readmission included five variables: narcotic use at the time of discharge, benzodiazepines given during admission, admission for pain, abscess drainage, and discharge to assisted home care or assisted care facility. A more recent assessment of risk factors for readmission included 356 patients with an IBD-related admission over a two-year study period at a single center. Depression, chronic pain and steroid use in the 6-months prior to index admission were related to 90-day readmissions [19].

The patients in our study had an overall rate of readmission of 7%, falling between the previously reported 30-day readmission rate among individuals with IBD from Nguyen *et al* (2.3%) [15] and Hazratjee *et al* (25.7%) [18], although considerably less than readmission rates for chronic conditions such as heart failure (30%) [20] and cirrhosis (12.9–24.2%) [21]. While not all risk factors for readmissions identified in this study or in previous investigations are modifiable, multiple studies have identified psychiatric comorbidities associated with increased 30-day readmission rates including anxiety, depression, and drug abuse in select populations [22]. While it is hypothesized that a concurrent psychiatric disorder of depression can lead to hopelessness, decreased motivation and self-care, and anxiety to result in heightened arousal and misinterpretation of symptoms, few studies address interventions to reduce readmissions [22]. Screening for symptoms of anxiety or depression does not consistently lead to a decreased rate of readmission [23], while utilization of novel telemonitoring has the potential to improve concurrent symptoms and hospitalization rates [24, 25].

Our study adds to the current literature with respect to factors associated with readmissions among individuals with IBD. We utilized a novel discharge database to create a large cohort of IBD patients assessing for risk factors related to readmission. While we were able to replicate the findings of previous studies to include patient age, disease type and psychiatric comorbidities, we were also able to identify novel factors related to health insurance, hospital volume and socioeconomic status. Self-paying patients, and those with private insurance were demonstrated to have a decreased risk of readmission when compared to individuals with Medicare, although likely for differing underlying explanations. Individuals with private insurance are hypothesized to have increased access to preventive post-discharge care leading to decreased rates of readmission, while self-paying individuals may avoid costly healthcare services such as rehospitalization [26]. With the introduction of the Affordable Care Act (ACA) increased coverage and access to care could likewise either decrease readmission rates through increased preventive services, or increase readmission rates through the encouragement to seek care [26].

Two recent studies have utilized the NRD for an assessment of readmission risk factors in a population with UC [27] and IBD [28]. While our study has similar findings with respect to the study by Barnes et al., to include multivariate factors related to readmission in CD (depression and chronic pain/opiate use) differences in the multivariate findings related to UC (a lack of association with depression or anxiety) are likely related to the differences in readmission definition (30 days versus 90 days) [28]. Our study has some similarities to the study by Poojary et al., with respect to age associations with 30-day readmission in UC; however, we found an increased risk of readmission with the performance of lower endoscopy as compared to the previous study in which an increased risk was demonstrated without the performance of endoscopy [27]. This difference is likely related to the baseline inclusion criteria in which our study excluded individuals with multiple hospital readmissions. Our study adds to the current literature in respect to utilization of previously reported inclusion criteria as well as creating simplified models of readmission risk within this database demonstrating that the use of only discharge diagnosis codes in determining readmission risk results in poor predictive ability.

A number of previous studies have provided important information evaluating IBD hospitalizations from the related database, the Nationwide Inpatient Sample (NIS), including: *C*. *difficile* infection [29] and hospital volume and mortality [14]. Utilizing the previous inclusion criteria, we were able to observe expected differences between CD and UC for rates of smoking, *C*. *difficile* colitis and stricture formation. Interestingly, individuals with UC had higher comparative rates of malnutrition and electrolyte disturbances compared to CD. This difference in unadjusted analysis was previously demonstrated using the NIS, although the difference was eliminated in an adjusted analysis to include age and comorbidity [30]. Utilizing a nationwide representative sample allows for an increased sample size to define risk factors, which otherwise is a limitation of most single center cohort studies. Furthermore, the unique variables in the database permitted us to explore factors such as hospitalization cost, household income estimates and hospital factors, which are not commonly available in single center studies.

Although we were able to demonstrate multiple risk factors for hospital readmission, the study has several limitations. Since the unit of analysis in the NRD is hospital discharges, we are unable to extract patient specific data related to disease phenotype. While we used previously described inclusion criteria to define hospitalizations among individuals with IBD within the NIS [13, 14], the absence of patient level data does not allow for verification of the accuracy of the ICD-9-CM coding. Factors such as medication use, outpatient medical utilization and objective laboratory values are not included in this analysis and may contribute to important associations related to the risk of readmission in IBD. The lack of details with respect to medication use hampers our ability to recommend specific interventions aimed at decreasing risk of readmission. While we found that individuals with a diagnosis of *C*. *difficile* and CD had a higher risk of readmission, we were unable to assess for differences in treatment course and readmission risk as demonstrated by Horton *et al*, in which treatment of *C*. *difficile* with vancomycin in UC led to a decreased risk of readmission [31]. Furthermore, as the NRD utilizes the SID to identify readmissions only within the same state, readmissions to hospitals not included in the SID or to out-of-state hospitals are not integrated in the NRD, and thus in the readmission analysis. Lastly, we are unable to assess for risk factors such as disease duration, disease severity, race, previous surgical procedures or previous utilization of inpatient medical care given the limited factors contained within the NRD. As this is the first release of readmission data in the NRD, future studies will allow for examination of readmission rates over prolonged periods of time.

In conclusion, this is the first analysis of a novel nationwide discharge database to assess for risk factors associated with 30-day readmission in IBD. We demonstrated important risk factors for 30-day readmission among individuals with IBD, highlighting the need for targeted interventions in order to decrease rates of readmission within this population. Despite the inherent limitations of an administrative database, the results of this investigation have many important clinical implications. Our primary findings include lower readmission rates among females, older patients, self-paying patients, and those with private insurance and in higher quartiles of household income. While these risk factors are not modifiable, focusing on the timely performance of surgery in Crohn’s disease, as well as identifying patients with concurrent comorbidities of anxiety and opioid dependence has the potential to decrease readmissions. Future studies focused on interventions in high-risk individuals with IBD have the potential to improve readmission rates.

## Supporting information

S1 Table Diagnosis and procedures codes. Included ICD-9 codes utilized for definitions within the analysis.
(DOCX)

S2 Table Ulcerative colitis risk factors for 30-day readmission. Univariate and multivariate risk factors for 30-day readmission among individuals with ulcerative colitis.
(DOCX)

S3 Table Crohn's disease risk factors for 30-day readmission. Univariate and multivariate risk factors for 30-day readmission among individuals with Crohn’s disease.
(DOCX)

S4 Table Surgery patient risk factors for 30-day readmission. Univariate and multivariate risk factors for 30-day readmission among individuals undergoing surgery.
(DOCX)

S1 Fig AUROC curve for all IBD readmission. Inclusion of opioid dependence, malnutrition, age > 65 and undergoing surgery had an AUROC of 0.58 with an optimal cut off of 0.07 with a sensitivity of 82% and specificity of 26%.
(JPG)

S2 Fig AUROC curve for Crohn’s disease readmission. Inclusion of opioid dependence, cannabis dependence, malnutrition and undergoing surgery produced an AUROC of 0.54 with an optimal cut off of 0.09 with a sensitivity of 15% and specificity of 91%
(JPG)

S3 Fig AUROC curve for ulcerative colitis readmission. Inclusion of age > 65, bowel obstruction, malnutrition and undergoing surgery produced an AUROC of 0.59 with an optimal cut off of 0.06 with a sensitivity of 80% and a specificity of 31%.
(JPG)

## Abbreviations

CD: Crohn’s disease
UC: Ulcerative colitis
IBD: Inflammatory bowel disease
HCUP: Healthcare Cost and Utilization Project
NRD: Nationwide Readmissions Database
AHRQ: Agency for Healthcare Research and Quality
SID: State Inpatient Databases
NS: not significant
ref: reference
HR: hazard ratio
