# Supplementary material for: Factors associated with readmission to the hospital within 30 days in patients with inflammatory bowel disease
Source: PLoS One. 2017 Aug 24;12(8):e0182900. doi: 10.1371/journal.pone.0182900 (PMC5570509; doi:10.1371/journal.pone.0182900)
Supplement: S1 Table — Included ICD-9 codes utilized for definitions within the analysis. (DOCX) [file pone.0182900.s001.docx]

**S1 Table: Diagnosis and procedures codes**

| **IBD-related complications:** | **Codes:** |
| --- | --- |
| Active fistulizing disease or intraabdominal abscess | ICD-9-CM codes 537.4, 567.2, 567.21, 567.22, 569.5, 569.81, 569.83, 593.3, 596.1 and 619.1 |
| Stricturing bowel disease | ICD-9-CM codes 560.9 and 537.3 |
| Other bowel obstruction | ICD-9-CM codes 560.x and 568.0 |
| Perianal abscess | ICD-9-CM code 566 |
| Unspecified lower gastrointestinal (GI) hemorrhage | ICD-9-CM codes 578.9 and 569.3 |
| **Systemic complications of IBD:** |  |
| Hypovolemia | ICD-9-CM code 276.5x |
| Electrolyte imbalance | ICD-9-CM codes 276.1 and 276.8 |
| Anemia | ICD-9-CM codes 280, 280.1, 280.9, 285.1 and 285.9 |
| Malnutrition | ICD-9-CM code 263.x |
| **Patient specific comorbidities:** |  |
| Opioid dependence | ICD-9-CM codes 304.0[0-2], 305.5[0-2] |
| Cannabis dependence | ICD-9-CM codes 304.3[0-2], 305.5[0-2] |
| Smoking | ICD-9-CM code 305.1 |
| Depression | CCS code 657 |
| Anxiety | CCS code 651 |
| **Procedures:** |  |
| Lower gastrointestinal (GI) endoscopy (sigmoidoscopy/colonoscopy) | ICD-9-CM codes 45.24, 48.23; 45.23, 45.25, 45.22, 48.24 |
| Blood product transfusion | ICD-9-CM codes 990.x |
| Computed tomography of the abdomen and pelvis | ICD-9-CM code 88.01 |
| Parenteral nutrition | ICD-9-CM code 99.15 |
| **Surgical procedures:** |  |
| Small bowel resection | ICD-9-CM codes 45.64, 45.61, 45.62, 45.63, 45.33 |
| Small bowel anastomosis | ICD-9-CM codes 45.90, 45.91, 45.5, 45.50, 45.51 |
| Partial or total colectomy | ICD-9-CM codes 45.7x, 45.8 |
| Large bowel anastomosis | ICD-9-CM codes 45.92-45.95 |

Hospital volume within the NRD is coded as small, medium or large and is dependent on the hospital region and urban-rural designation (1). Clinical Classification Software codes (CCS) that truncate ICD-9 codes into categories by diagnosis were used to obtain the diagnoses of depression and anxiety (2). Severity of illness and comorbidity status were determined by a proprietary ICD-9-CM-based algorithm made available through the AHRQ, referred to as the All-Patient Refined Diagnosis-Related Group (APR-DRG) risk of mortality and severity of illness scores (3M Health Systems, St. Paul, MN, USA) (3). A surgical procedure was considered to be elective if it was performed within 1 day of admission (day 0 or day 1), the admission was not from the emergency room, and the admission was characterized as elective (4).

**References**

1. Introduction to the HCUP Natiowide Readmissions Database (NRD) 2013. Available from: <https://www.hcup-us.ahrq.gov/nrdoverview.jsp>.

2. Clinical Classifications Software (CCS) for ICD-9-CM [updated 4/14/16]. Available from: <http://www.hcup-us.ahrq.gov/toolssoftware/ccs/ccs.jsp>.

3. Shen Y. Applying the 3M All Patient Refined Diagnosis Related Groups Grouper to measure inpatient severity in the VA. Medical care. 2003;41(6 Suppl):Ii103-10.

4. Ananthakrishnan AN, McGinley EL, Binion DG. Does it matter where you are hospitalized for inflammatory bowel disease? A nationwide analysis of hospital volume. The American journal of gastroenterology. 2008;103(11):2789-98.
