# Supplementary material for: Factors associated with readmission to the hospital within 30 days in patients with inflammatory bowel disease
Source: PLoS One. 2017 Aug 24;12(8):e0182900. doi: 10.1371/journal.pone.0182900 (PMC5570509; doi:10.1371/journal.pone.0182900)
Supplement: S2 Table — Univariate and multivariate risk factors for 30-day readmission among individuals with ulcerative colitis. (DOCX) [file pone.0182900.s002.docx]

**S2 Table: Ulcerative colitis Risk Factors for 30-day Readmission**

| Characteristic | Not readmitted within 30 days (n = 15,817) | Readmitted within 30 days (n = 1091) | *Univariate P-*value | Multivariate OR (95% CI) |
| --- | --- | --- | --- | --- |
| Mean age (years) | 52.2 | 47.3 |  |  |
| Age group (years) |  |  |  |  |
| 18-35 | 4104 (25.9%) | 387 (35.5%) | <0.001 | **Ref** |
| 36-50 | 3283 (20.8%) | 250 (22.9%) | 0.09 | **.84 (.71-.99)** |
| 51-65 | 3868 (24.5%) | 233 (21.3%) | 0.021 | **.66 (.55-.78)** |
| >65 | 4562 (28.8%) | 221 (20.2%) | <0.001 | **.51 (.39-.65)** |
| Sex |  |  |  |  |
| Male | 7230 (45.7%) | 549 (50.4%) | 0.003 | **1.19 (1.05-1.35)** |
| Female | 8587 (54.3%) | 542 (49.6%) | 0.003 | **Ref** |
| APR-DRG Risk of Mortality |  |  |  |  |
| Minor | 9850 (62.3%) | 728 (66.7%) | 0.003 |  |
| Moderate | 3328 (21.0%) | 225 (20.6%) | 0.743 |  |
| Major | 2058 (13.0%) | 121 (11.2%) | 0.067 |  |
| Extreme | 580 (3.7%) | 17 (1.6%) | <0.001 |  |
| Smoking | 3313 (20.9%) | 234 (21.4%) | 0.693 |  |
| Depression | 2337 (14.8%) | 163 (15.0%) | 0.882 |  |
| Anxiety | 1724 (10.9%) | 133 (12.2%) | 0.187 |  |
| Depression and Anxiety | 814 (5.1%) | 64 (5.9%) | 0.30 |  |
| Opioid dependence | 120 (0.8%) | 13 (1.2%) | 0.117 |  |
| Cannabis dependence | 136 (0.9%) | 7 (0.6%) | 0.447 |  |
| Weekend admission | 3341 (21.1%) | 210 (19.2%) | 0.142 |  |
| Length of stay (mean days) | 5.8 | 6 | 0.238 |  |
| Total charges (mean USD) | 47,106 | 42,389 | 0.041 |  |
| Primary payer |  |  |  |  |
| Medicare | 5462 (34.6%) | 288 (26.5%) | <0.001 | **Ref** |
| Medicaid | 1735 (11.0%) | 140 (12.8%) | 0.058 | **NS** |
| Private | 6804 (43.1%) | 532 (48.7%) | <0.001 | **NS** |
| Self pay | 934 (5.9%) | 65 (6.0%) | 0.943 | **NS** |
| No charge | 135 (0.9%) | 18 (1.6%) | 0.007 | **NS** |
| Other | 729 (4.6%) | 48 (4.4%) | 0.749 | **NS** |
| Median income quartiles for patient’s ZIP code |  |  |  |  |
| Quartile 1 (lowest income) | 3396 (21.8%) | 246 (23.0%) | 0.402 |  |
| Quartile 2 | 3789 (24.3%) | 258 (24.1%) | 0.818 |  |
| Quartile 3 | 4133 (26.5%) | 283 (26.5%) | 0.89 |  |
| Quartile 4 (highest income) | 4272 (27.4%) | 283 (26.4%) | 0.441 |  |
| Teaching status of hospitals |  |  |  |  |
| Metropolitan non-teaching | 6524 (41.2%) | 437 (40.0%) | 0.439 |  |
| Metropolitan teaching | 8148 (51.5%) | 592 (54.3%) | 0.079 |  |
| Non-metropolitan | 1145 (7.2%) | 62 (5.7%) | 0.053 |  |
| Hospital volume |  |  |  |  |
| Low | 1679 (10.6%) | 115 (10.5%) | 0.939 |  |
| Medium | 3710 (23.5%) | 276 (25.3%) | 0.166 |  |
| High | 10,428 (65.9%) | 700 (64.2%) | 0.234 |  |
| Disease complications |  |  |  |  |
| Stricture | 614 (3.9%) | 26 (2.4%) | 0.012 | **NS** |
| Bowel obstruction | 869 (5.5%) | 36 (3.3%) | 0.002 | **.69 (.48-.97)** |
| Gastrointestinal bleeding | 1,211 (7.7%) | 75 (7.0%) | 0.346 |  |
| *Clostridium difficile* colitis | 1620 (10.2%) | 117 (10.7%) | 0.612 |  |
| Hypovolemia | 3064 (19.4%) | 246 (22.5%) | 0.011 | **1.21 (1.04-1.41)** |
| Electrolyte disturbance | 5095 (32.2%) | 381 (35.0%) | 0.064 |  |
| Anemia | 1837 (11.6%) | 156 (14.3%) | 0.008 | **NS** |
| Malnutrition | 1590 (10.1%) | 160 (14.7%) | <0.001 | **1.59 (1.33 - 1.90)** |
| Hospitalization characteristics |  |  |  |  |
| Lower endoscopy | 5284 (33.4%) | 423 (38.7%) | <0.001 | **1.15 (1.01-1.31)** |
| Abdominal CT scan | 647 (4.1%) | 38 (3.5%) | 0.325 |  |
| Blood transfusion | 2215 (14.0%) | 170 (15.7%) | 0.148 |  |
| Small bowel resection | 142 (0.9%) | 5 (0.5%) | 0.13 |  |
| Colectomy (partial or total) | 629 (4.0%) | 27 (2.5%) | 0.013 |  |
| Any surgery performed | 732 (4.6%) | 28 (2.6%) | 0.001 | **NS** |
| Elective surgery | 529 (3.3%) | 20 (1.8%) | 0.006 | **NS** |
