# Supplementary material for: Factors associated with readmission to the hospital within 30 days in patients with inflammatory bowel disease
Source: PLoS One. 2017 Aug 24;12(8):e0182900. doi: 10.1371/journal.pone.0182900 (PMC5570509; doi:10.1371/journal.pone.0182900)
Supplement: S3 Table — Univariate and multivariate risk factors for 30-day readmission among individuals with Crohn’s disease. (DOCX) [file pone.0182900.s003.docx]

**S3 Table: Crohn's disease Risk Factors for 30-day Readmission**

| Characteristic | Not readmitted within 30 days (n = 24,826) | Readmitted within 30 days (n = 1944) | *Univariate P-*value | Multivariate OR (95% CI) |
| --- | --- | --- | --- | --- |
| Mean age (years) | 47 | 43.7 |  |  |
| Age group (years) |  |  |  |  |
| 18-35 | 7949 (32.0%) | 82009 (42.2%) | <0.001 | **Ref** |
| 36-50 | 6436 (25.9%) | 486 (25.0%) | 0.370 | **0.71 (0.63-0.8)** |
| 51-65 | 5714 (23.0%) | 358 (18.5%) | <0.001 | **0.57 (0.5-0.66)** |
| >65 | 4727 (19.0%) | 279 (14.4%) | <0.001 | **0.45 (0.38-0.55)** |
| Sex |  |  |  |  |
| Male | 10,702 (43.1%) | 880 (45.3%) | 0.064 | **1.11 (1.01-1.22)** |
| Female | 14,124 (56.9%) | 1063 (54.7%) | 0.064 | **Ref** |
| APR-DRG Risk of Mortality |  |  |  |  |
| Minor | 16,264 (65.5%) | 1278 (65.8%) | 0.802 |  |
| Moderate | 5274 (21.2%) | 413 (21.2%) | 0.999 |  |
| Major | 2620 (10.6%) | 209 (10.8%) | 0.731 |  |
| Extreme | 668 (2.7%) | 42 (2.2%) | 0.161 |  |
| Smoking | 6987 (28.1%) | 616 (31.7%) | 0.001 | **NS** |
| Depression | 4161 (16.8%) | 394 (20.3%) | <0.001 | **1.16 (1.01-1.35)** |
| Anxiety | 3130 (12.6%) | 303 (15.6) | <0.001 | **NS** |
| Depression and Anxiety | 1528 (6.2%) | 157 (8.1%) | 0.001 | **NS** |
| Opioid dependence | 333 (1.3%) | 47 (2.4%) | <0.001 | **1.38 (1.01-1.9)** |
| Cannabis dependence | 338 (1.4%) | 41 (2.1%) | 0.01 | **NS** |
| Weekend admission | 5147 (20.7%) | 431 (22.2%) | 0.133 |  |
| Length of stay (mean days) | 5.1 | 5.7 | <0.001 |  |
| Total charges (mean USD) | 40,778 | 42,084 | 0.374 |  |
| Primary payer |  |  |  |  |
| Medicare | 7450 (30.0%) | 571 (29.4%) | 0.588 | **Ref** |
| Medicaid | 3143 (12.7%) | 369 (19%) | <0.001 | **NS** |
| Private | 11,175 (45.1%) | 725 (37.3%) | <0.001 | **0.67 (0.58-0.77)** |
| Self pay | 1719 (6.9%) | 157 (8.1%) | 0.045 | **NS** |
| No charge | 269 (1.1%) | 23 (1.2%) | 0.684 | **NS** |
| Other | 1040 (4.2%) | 93 (4.8%) | 0.210 | **NS** |
| Median income quartiles for patient’s ZIP code |  |  |  | **NS** |
| Quartile 1 (lowest income) | 5518 (22.6%) | 516 (26.5%) | <0.001 | **Ref** |
| Quartile 2 | 6406 (26.2%) | 498 (25.6%) | 0.857 | **NS** |
| Quartile 3 | 6361 (26.0%) | 467 (24%) | 0.119 | **0.86 (0.75-0.98)** |
| Quartile 4 (highest income) | 6157 (25.2%) | 439 (22.6%) | 0.029 | **NS** |
| Teaching status of hospitals |  |  |  |  |
| Metropolitan non-teaching | 10,039 (40.4%) | 752 (38.7%) | 0.129 | **0.76 (0.62-0.92)** |
| Metropolitan teaching | 12,739 (51.3%) | 1062 (54.6%) | 0.005 | **Ref** |
| Non-metropolitan | 2048 (8.2%) | 130 (6.7%) | 0.015 | **NS** |
| Hospital volume |  |  |  |  |
| Low | 2721 (11.0%) | 170 (8.8%) | 0.003 | **Ref** |
| Medium | 5975 (24.1%) | 482 (24.8%) | 0.471 | **1.25 (1.04-1.51)** |
| High | 16,130 (65.0%) | 1291 (66.4%) | 0.201 | **1.21 (1.02-1.43)** |
| Disease complications |  |  |  |  |
| Intraabdominal fistula or abscess | 2830 (11.4%) | 274 (14.1%) | <0.001 | **1.33 (1.15-1.53)** |
| Perianal fistula or abscess | 866 (3.5%) | 75 (3.9%) | 0.394 |  |
| Stricture | 3928 (15.8%) | 284 (14.6%) | 0.157 |  |
| Bowel obstruction | 2027 (8.2%) | 137 (7.0%) | 0.082 |  |
| Gastrointestinal bleeding | 1317 (5.3%) | 108 (5.6%) | 0.635 |  |
| *Clostridium difficile* colitis | 942 (3.8%) | 103 (5.3%) | 0.001 | **1.25 (1.01-1.55)** |
| Hypovolemia | 3655 (14.7%) | 340 (17.5%) | 0.001 | **1.22 (1.07-1.38)** |
| Electrolyte disturbance | 6410 (25.8%) | 560 (28.8%) | 0.004 | **1.13 (1.02-1.26)** |
| Anemia | 2186 (8.8%) | 178 (9.2%) | 0.599 |  |
| Malnutrition | 2160 (8.7%) | 233 (12.0%) | <0.001 | **1.26 (1.08-1.47)** |
| Hospitalization characteristics |  |  |  |  |
| Lower endoscopy | 4401 (17.7%) | 321 (16.6%) | 0.195 |  |
| Abdominal CT scan | 1002 (4.0%) | 72 (3.7%) | 0.472 |  |
| Blood transfusion | 2223 (9.0%) | 209 (10.7%) | 0.008 | **1.26 (1.08-1.48)** |
| Small bowel resection | 912 (3.7%) | 34 (1.7%) | <0.001 |  |
| Colectomy (partial or total) | 1649 (6.6%) | 80 (4.1%) | <0.001 |  |
| Any surgery performed | 2272 (9.2%) | 99 (5.1%) | <0.001 | **0.54 (0.3-0.99)** |
| Elective surgery | 1212 (4.9%) | 46 (2.4%) | <0.001 | **NS** |
| Urgent surgery | 844 (3.4%) | 42 (2.2%) | 0.003 | **NS** |
