# Supplementary material for: Factors associated with readmission to the hospital within 30 days in patients with inflammatory bowel disease
Source: PLoS One. 2017 Aug 24;12(8):e0182900. doi: 10.1371/journal.pone.0182900 (PMC5570509; doi:10.1371/journal.pone.0182900)
Supplement: S4 Table — Univariate and multivariate risk factors for 30-day readmission among individuals undergoing surgery. (DOCX) [file pone.0182900.s004.docx]

**S4 Table: Surgery Patient Risk Factors for 30-day Readmission**

| Characteristic | Not readmitted within 30 days (n = 3004) | Readmitted within 30 days (n = 127) | *Univariate P-*value | Multivariate OR (95% CI) |
| --- | --- | --- | --- | --- |
| Mean age (years) | 46.6 | 44.9 |  |  |
| Crohn's disease | 2272 (75.6%) | 99 (78.0%) | 0.55 |  |
| Ulcerative colitis | 732 (24.4%) | 28 (22.0%) | 0.55 |  |
| Age group (years) |  |  |  |  |
| 18-35 | 945 (31.5%0 | 45 (35.4%) | 0.346 | **Ref** |
| 36-50 | 813 (27.1%) | 34 (26.8%) | 0.942 | **NS** |
| 51-65 | 766 (25.5%) | 31 (24.4%) | 0.782 | **NS** |
| >65 | 480 (16.0%) | 17 (13.4%) | 0.434 | **NS** |
| Sex |  |  |  |  |
| Male | 1516 (50.5%) | 60 (47.2%) | 0.477 | **NS** |
| Female | 1488 (49.5%) | 67 (52.8%) | 0.477 | **Ref** |
| APR-DRG Risk of Mortality |  |  |  |  |
| Minor | 1685 (56.1%) | 62 (48.8%) | 0.106 |  |
| Moderate | 648 (21.6%) | 20 (15.7%) | 0.117 |  |
| Major | 471 (15.7%) | 27 (21.3%) | 0.092 |  |
| Extreme | 200 (6.7%) | 18 (14.2%) | 0.001 |  |
| Smoking | 734 (24.4%) | 40 (31.5%) | 0.071 |  |
| Depression | 373 (12.4%) | 22 (17.3%) | 0.103 |  |
| Anxiety | 316 (10.5%) | 18 (14.2%) | 0.191 |  |
| Depression and Anxiety | 142 (4.7%) | 8 (6.3%) | 0.417 |  |
| Opioid dependence | 23 (0.8%) | 0 (0%) | 0.322 |  |
| Cannabis dependence | 17 (0.6%) | 1 (0.8%) | 0.746 |  |
| Weekend admission | 266 (8.9%) | 16 (12.6%) | 0.149 |  |
| Length of stay (mean days) | 10 | 13.7 | <0.001 |  |
| Total charges (mean USD) | 96,994 | 127,562 | 0.01 |  |
| Primary payer |  |  |  |  |
| Medicare | 694 (23.1%) | 35 (27.6%) | 0.245 |  |
| Medicaid | 263 (8.8%) | 17 (13.4%) | 0.073 |  |
| Private | 1769 (59.0%) | 67 (52.8%) | 0.169 |  |
| Self pay | 123 (4.1%) | 3 (2.4%) | 0.331 |  |
| No charge | 14 (0.5%) | 1 (0.8%) | 0.608 |  |
| Other | 137 (4.6%) | 4 (3.1%) | 0.453 |  |
| Median income quartiles for patient’s ZIP code |  |  |  |  |
| Quartile 1 (lowest income) | 569 (19.2%) | 33 (26.2%) | 0.049 |  |
| Quartile 2 | 771 (26.1%) | 29 (23.0%) | 0.474 |  |
| Quartile 3 | 784 (26.5%) | 36 (28.6%) | 0.573 |  |
| Quartile 4 (highest income) | 833 (28.2%) | 28 (22.2%) | 0.160 |  |
| Teaching status of hospitals |  |  |  |  |
| Metropolitan non-teaching | 892 (29.7%) | 34 (26.8%) | 0.480 |  |
| Metropolitan teaching | 1981 (65.9%) | 88 (69.3%) | 0.435 |  |
| Non-metropolitan | 131 (4.4%0 | 5 (3.9%) | 0.819 |  |
| Hospital volume |  |  |  |  |
| Low | 278 (9.3%) | 9 (7.1%) | 0.407 |  |
| Medium | 625 (20.8%) | 37 (29.1%) | 0.024 |  |
| High | 2101 (69.9%) | 81 (63.8%) | 0.139 |  |
| Disease complications |  |  |  |  |
| Intraabdominal fistula or abscess | 888 (29.6%) | 41 (32.3%) | 0.511 |  |
| Perianal fistula or abscess | 27 (0.9%) | 3 (2.4%) | 0.097 |  |
| Stricture | 467 (15.5%) | 25 (19.7%) | 0.209 |  |
| Bowel obstruction | 1043 (34.7%) | 55 (43.3%) | 0.047 | **NS** |
| Gastrointestinal bleeding | 72 (2.4%0 | 5 (3.9%) | 0.272 |  |
| *Clostridium difficile* colitis | 84 (2.8%) | 2 (1.6%) | 0.410 |  |
| Hypovolemia | 189 (6.3%) | 17 (13.4%) | 0.002 | **1.86 (1.07-3.22)** |
| Electrolyte disturbance | 672 (22.4%) | 31 (24.4%) | 0.59 |  |
| Anemia | 178 (5.9%) | 15 (11.8%) | 0.007 | **1.9 (1.07-3.38)** |
| Malnutrition | 442 (14.7%) | 31 (24.4%) | 0.003 | **1.56 (1.01-2.42)** |
| Hospitalization characteristics |  |  |  |  |
| Lower endoscopy | 356 (11.9%) | 19 (15.0%) | 0.29 |  |
| Abdominal CT scan | 73 (2.4%) | 2 (1.6%) | 0.537 |  |
| Blood transfusion | 415 (13.8%) | 34 (26.8%) | <0.001 | **1.96 (1.27-3.02)** |
| Small bowel resection | 1054 (35.1%) | 39 (30.7%) | 0.311 |  |
| Colectomy (partial or total) | 2278 (75.8%) | 107 (84.3%) | 0.029 | **1.84 (1.3-3.0)** |
| Elective surgery | 1741 (58.0%) | 66 (52.0%) | 0.181 |  |
| Urgent surgery | 991 (33.0%) | 47 (37.0%) | 0.346 |  |
