# Supplementary figures and images for: Factors associated with readmission to the hospital within 30 days in patients with inflammatory bowel disease
Source: PLoS One. 2017 Aug 24;12(8):e0182900. doi: 10.1371/journal.pone.0182900 (PMC5570509; doi:10.1371/journal.pone.0182900)

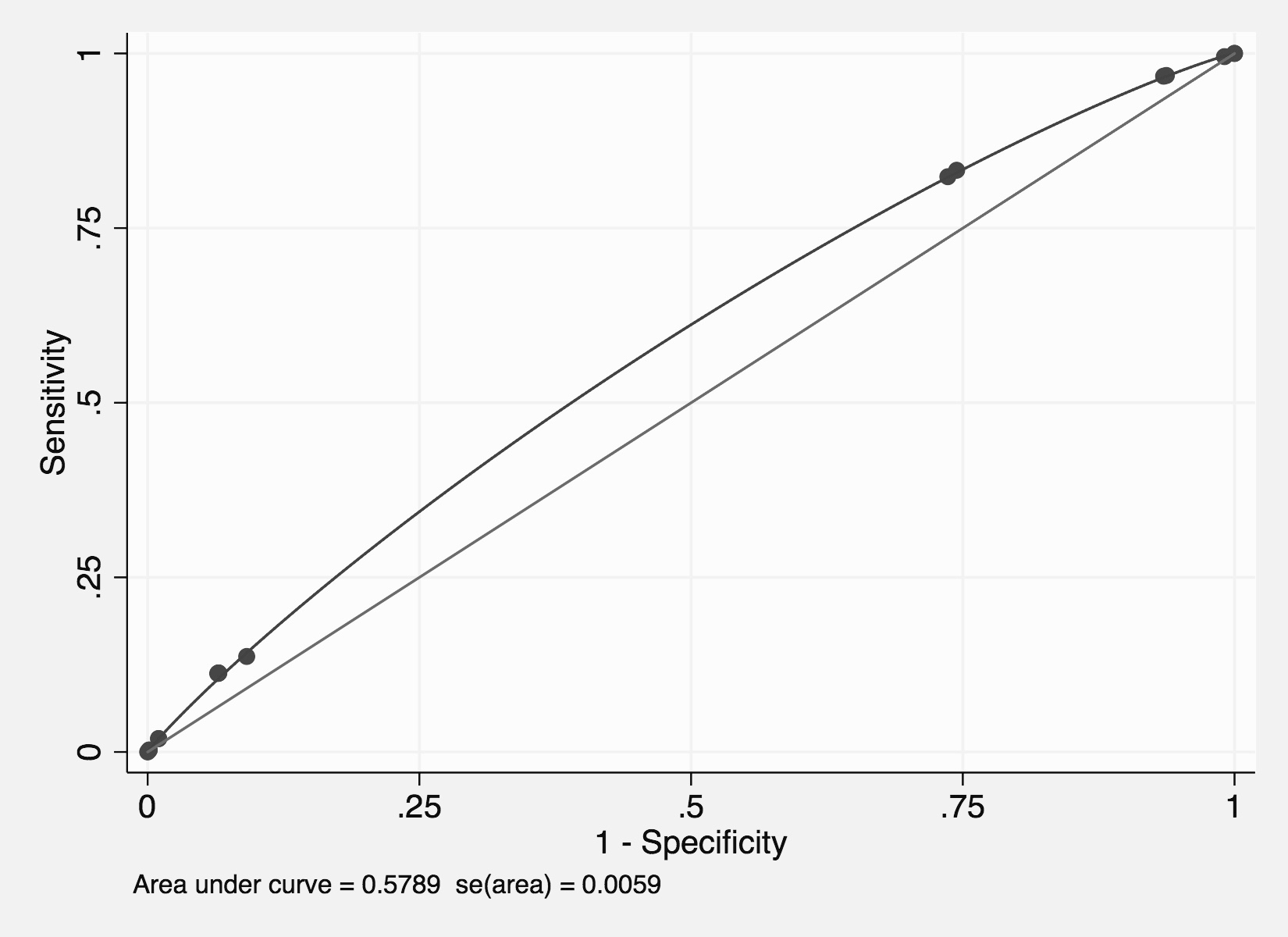

Supplement: S1 Fig — Inclusion of opioid dependence, malnutrition, age > 65 and undergoing surgery had an AUROC of 0.58 with an optimal cut off of 0.07 with a sensitivity of 82% and specificity of 26%. (JPG) [file pone.0182900.s005.jpg]

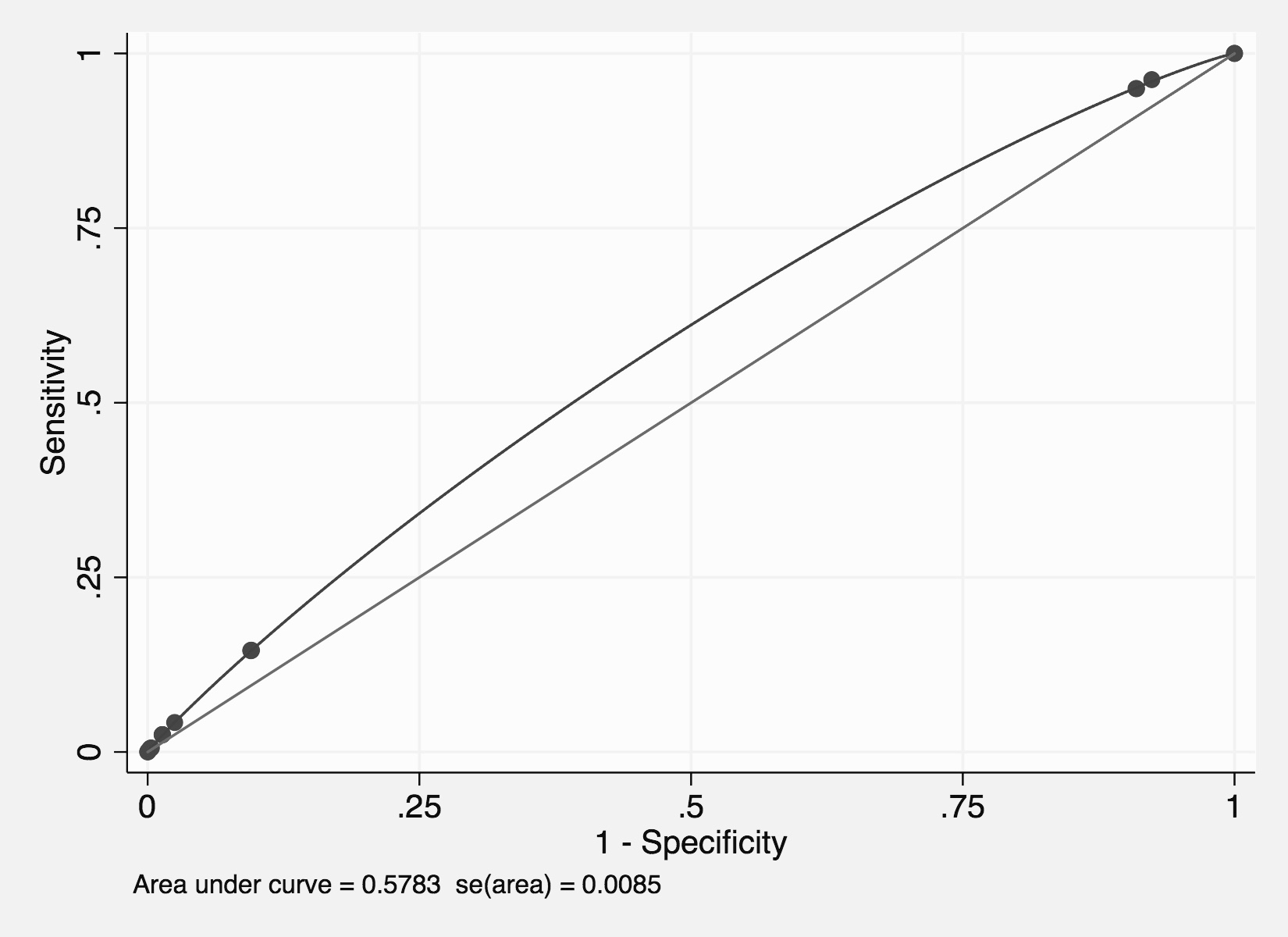

Supplement: S2 Fig — Inclusion of opioid dependence, cannabis dependence, malnutrition and undergoing surgery produced an AUROC of 0.54 with an optimal cut off of 0.09 with a sensitivity of 15% and specificity of 91% (JPG) [file pone.0182900.s006.jpg]

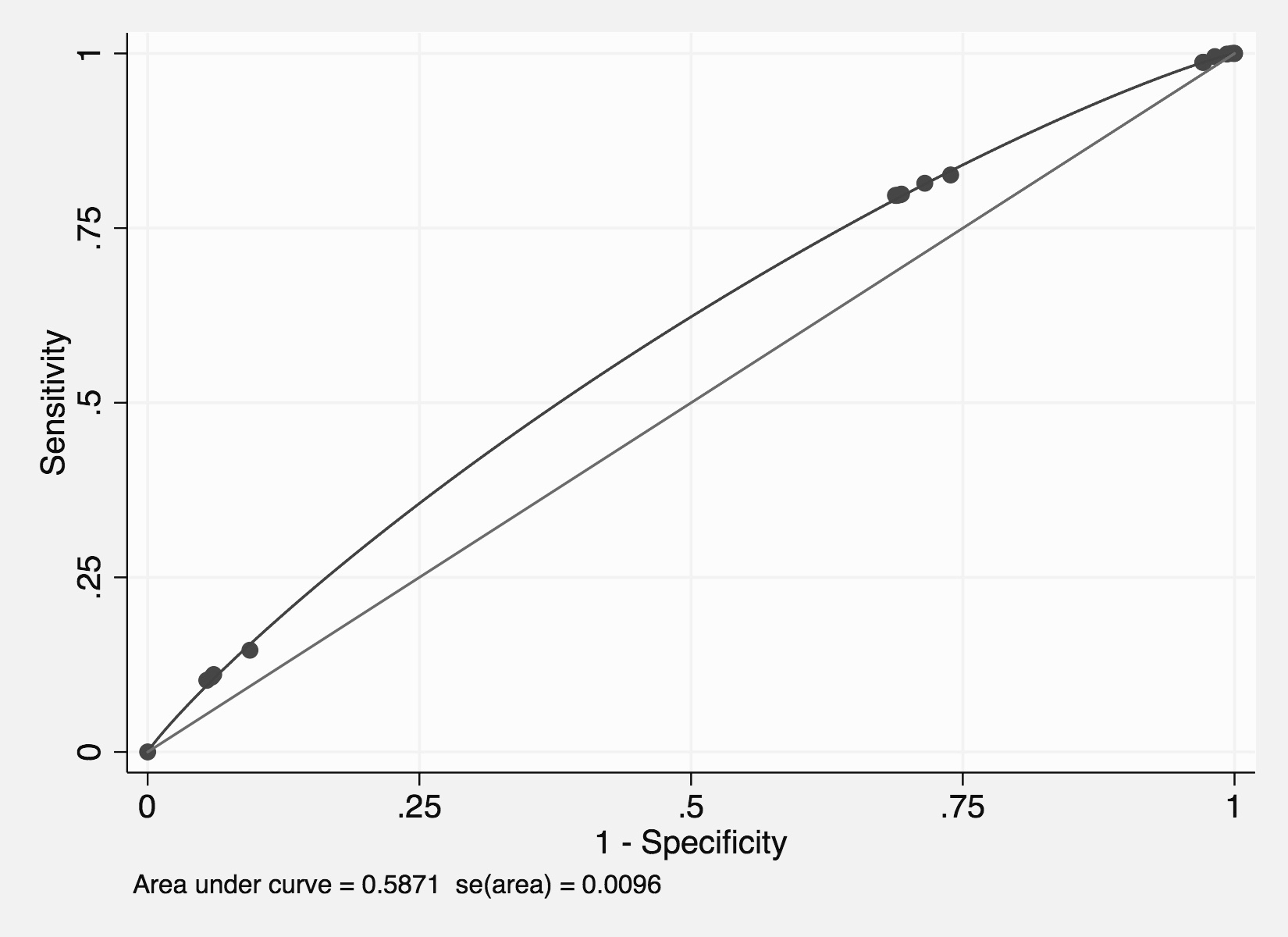

Supplement: S3 Fig — Inclusion of age > 65, bowel obstruction, malnutrition and undergoing surgery produced an AUROC of 0.59 with an optimal cut off of 0.06 with a sensitivity of 80% and a specificity of 31%. (JPG) [file pone.0182900.s007.jpg]
